# The transparency of quantitative empirical legal research published in highly ranked law journals (2018–2020): an observational study

**DOI:** 10.12688/f1000research.127563.2

**Published:** 2024-03-07

**Authors:** Jason Chin, Kathryn Zeiler, Natali Dilevski, Alex Holcombe, Rosemary Gatfield-Jeffries, Ruby Bishop, Simine Vazire, Sarah Schiavone

**Affiliations:** 1 College of Law, Australian National University, Canberra, ACT, Australia; 2 School of Law, University of Boston, Boston, MA, USA; 3 Centre for Investigative Interviewing, Griffith Criminology Institute, Griffith University, Brisbane, Qld, Australia; 4 Psychology, University of Sydney, Sydney, NSW, Australia; 5 History and Philosophy of Science, University of Cambridge, Cambridge, UK; 6 School of Law, University of Sydney, Sydney, NSW, Australia; 7 Melbourne School of Psychological Sciences, University of Melbourne, Melbourne, Vic, Australia; 8 Psychology, University of California, Davis, Davis, CA, USA

**Keywords:** metaresearch, open science, transparency, credibility, empirical legal research

## Abstract

**Background:**

Scientists are increasingly concerned with making their work easy to verify and build upon. Associated practices include sharing data, materials, and analytic scripts, and preregistering protocols. This shift towards increased transparency and rigor has been referred to as a “credibility revolution.” The credibility of empirical legal research has been questioned in the past due to its distinctive peer review system and because the legal background of its researchers means that many often are not trained in study design or statistics. Still, there has been no systematic study of transparency and credibility-related characteristics of published empirical legal research.

**Methods:**

To fill this gap and provide an estimate of current practices that can be tracked as the field evolves, we assessed 300 empirical articles from highly ranked law journals including both faculty-edited journals and student-edited journals.

**Results:**

We found high levels of article accessibility (86%, 95% CI = [82%, 90%]), especially among student-edited journals (100%). Few articles stated that a study’s data are available (19%, 95% CI = [15%, 23%]). Statements of preregistration (3%, 95% CI = [1%, 5%]) and availability of analytic scripts (6%, 95% CI = [4%, 9%]) were very uncommon. (i.e., they collected new data using the study’s reported methods, but found results inconsistent or not as strong as the original).

**Conclusion:**

We suggest that empirical legal researchers and the journals that publish their work cultivate norms and practices to encourage research credibility. Our estimates may be revisited to track the field’s progress in the coming years.

## Introduction

Increasing the transparency of research is a key component of the ongoing credibility revolution ^1^ occurring in many fields. ^2^ This movement seeks to improve research credibility by ensuring that claims can be tested and critiqued by other researchers. Further benefits of the credibility revolution are efficiency, in that transparent research is reusable by other researchers to explore new questions, ^3^ and that transparent research enhances public trust in science, comporting with lay expectations about how science ought to be conducted. ^4^ Despite its work being cited by courts and policymakers, ^5^ the field of empirical legal research has so far largely refrained from engaging in significant reforms. In this article, we measure the transparency and other related characteristics of 300 empirical legal studies published between 2018 and 2020 in law journals rated highly by traditional metrics. For the purposes of this article, we define empirical research as research that performs analysis on quantitative data. ^6^

### The credibility revolution and the role of transparency

The “credibility revolution” ^7^ responded, in part, to a “crisis” ^8^ reported in many fields, in which researchers were unable to replicate the findings of published studies (i.e., they collected new data using the study’s reported methods, but found results inconsistent or not as strong as the original). ^9^ Failures to replicate and other controversies were well-publicized and documented in psychology. ^10^ However, other fields that run adjacent to legal research have not been immune, such as economics ^11^ and criminology. ^12^ Recently, for instance, economists have described and documented reproducibility failures in studies employing secondary data. ^13^

The credibility revolution involves a host of changes to the research process, such as improved transparency, higher standards of evidence, and more replication research. ^14^

Transparency-focused reforms can make research more efficient because other researchers can leverage open data and materials to test new questions, and to synthesize existing data in meta-analyses. ^15^ Conversely, research efforts can be wasted in the absence of open data in the sense that those data cannot be obtained by subsequent researchers seeking to reuse them. This is because researchers change email addresses and institutions or leave academic research behind altogether, making them unavailable to share data upon request. ^16^ Moreover, many researchers who are reachable, decline to share data and materials when they are contacted, or promise to deliver the data but never follow through. ^17^

Transparency and fuller reporting in the form of data sharing, as well as providing more details of methods and statistical analyses performed, allows other researchers to better scrutinize findings and detect errors in research. ^18^ For instance, researchers recently discovered a case of data fraud in a study purporting to find that signing one’s name before versus after providing information in a document reduces dishonesty. ^19^ This study has been cited often for its legal and policy consequences, ^20^ including by the UK Behavioural Insights Team (i.e., Nudge Unit). ^21^ Beyond availability of the raw data, which helped other researchers to uncover the fraud, replication also played a role. Failures to replicate other studies in the paper led to increased scrutiny of the entire set of results, which eventually led researchers to take a closer look at the data. One of the authors of the problematic paper, who had worked on the non-fraudulent studies reported within the same article, wrote in response to the discovery of the fraud: ^22^

Though very painful, this experience has reinforced my strong commitment to the Open Science movement. **As it clearly shows, posting data publicly, pre-registering studies, and conducting replications of prior research is key to scientific progress**.

Note that this is a quote from Francesca Gino. When we wrote the first version of this article, Gino had not yet been accused of fraud in relation to other studies. ^23^ That second potential fraud attributed to Gino was also discovered by way of the underlying data being available.

In addition to data and analysis scripts (i.e., code that researchers feed into statistical software packages such as R and STATA to produce reported results), transparency is advanced through preregistration (or prospective trial registration and a pre-analysis plan, as it is called in medical research and economics respectively), which is a time-stamped statement of the research protocols and hypotheses that is posted prior to data collection. ^24^ Preregistration is designed to address publication bias (i.e., the tendency for journal editors to prefer studies that produce statistically significant results) and questionable research practices (i.e., practices that increase the likelihood of publication but decrease the likelihood of successful replication—e.g., producing results using many different empirical models and reporting only statistically significant results).

Similarly, registered reports aim to promote transparency and decrease incentives to engage in questionable research practices. ^25^ Registered reports are studies that begin with peer review of the research plan prior to data collection and are accepted or rejected based solely on the plan and whether the researcher, after collecting data, follows the plan, Early research suggests results from studies published using a registered report protocol contain a more realistic proportion of null results. ^26^

### Measuring transparency and credibility-related features of published research

Several metascientific studies, across a variety of fields, have conducted “state-of-the-science” audits, in which recent published studies are randomly sampled and coded for various transparency and credibility-related features. ^27^ These metascientific studies have generally found very low levels of transparency. One study examined psychology articles published from 2014-2017. ^28^ Only about 2% of the studies sampled had available data, approximately 17% had available materials, and 3% were preregistered. ^29^ Note, however, that studies published during this timeframe were conducted in the early days of the reported crisis in psychology. ^30^

While these findings are worrisome, recent reforms in other fields may have led to an increase in transparency related practices in recent years. For instance, journals that implemented open data policies (e.g., requiring open data under some circumstances) show substantial increases in the proportion of studies with open data, albeit with imperfect compliance. ^31^

Moreover, a survey across many fields directly asking researchers about when they first engaged in a transparency-related practice (open data, open materials, open code, and preregistration) found that uptake has increased in recent years, suggesting that recent reforms and initiatives are moving the needle. ^32^

### Empirical legal research

Numerous researchers have questioned the credibility of empirical legal research. In a relatively early critique, Epstein and King reviewed all law journal articles published over a ten-year period that contain the word “empirical” in the title. ^33^ They found numerous errors, generally centering around poor transparency and reproducibility. For instance, many authors had not fully described how they gathered data and then reasoned from that data to their conclusion. Similar critiques have been levied since then, such as reports that empirical legal studies misinterpret statistical results (e.g., p-values), misapply statistical methods, and fail to verify that the assumptions underlying their methods were met. ^34^ Furthermore, author eminence likely plays a biasing role in empirical legal research because student editors may be especially vulnerable to accepting articles based on the status of the author. Even outside of the student context, author status has been shown to affect peer review decisions. ^35^ Most recently, Huber and colleagues found that an article submitted with a Nobel Laureate as corresponding author received over 40% fewer reject recommendations as compared to the same manuscript with a PhD student as corresponding author. ^36^

Matthews and Rantanen conducted the most recent metaresearch on empirical legal research, measuring data availability. ^37^ They sampled from the top 20 journals in the Washington & Lee rankings from 2010-2019, as well the *Northwestern Law Review* and the *Journal of Empirical Legal Studies.* They added the latter two because they provided a contrast with the other journals in the sample in terms of peer review. The *Northwestern Law Review* is one of the rare student-edited journals to routinely seek peer reviews for empirical work, and the *Journal of Empirical Legal Studies* is fully faculty-edited and peer reviewed. Matthews and Rantanen found low levels of data availability across the 614 articles in their sample, with only 12% making data available without contacting the author. Moreover – and despite its specialization on empirical works and policy encouraging authors to make their data available – the *Journal of Empirical Legal Studies* underperformed the other journals with only 6% data availability. These results converge with a 2021 study finding that highly ranked law journals implemented almost no transparency guidelines or requirements.

Limited data availability is especially troubling given several other aspects of empirical legal research that sets it apart from cognate fields. For instance, as individuals formally trained in the law rather than in empirical science, many authors of empirical legal work have less methodological expertise than researchers in other sciences. This lack of training may contribute to errors and unfamiliarity with methodological safeguards. The field’s lack of expertise also limits the usefulness of peer review (for journals that do use it).

These factors suggest that transparency is especially important for empirical legal research. For instance, accessible data and analytic scripts and preregistration can assist with error and bias detection. And, other aspects of transparency, such as articles that are openly available and declare funding sources and conflict of interests, help others assign credibility to reported results. Still, outside of the low data availability at elite journals, there is little current knowledge about transparency of empirical legal research. The last large study that assessed a broad array of transparency indicia was conducted 20 years ago. It included only articles with “empirical” in the title ^38^ and the results were not quantified in a way that makes them easy to update and revisit. This study seeks to fill these gaps.

## Methods

### Overview and design

To estimate the transparency of credibility-related features of recent empirical legal research, we examined a sample of 300 law journal articles published between 2018 and 2020. We chose this sample size because it is consistent with many previous transparency studies. ^39^ Based on those authors’ reports ^40^ of how long it took them to extract the relevant features of each article, we judged that coding 300 articles was a practical target given our available resources. To provide a comparison between the student-edited journals (that tend to not use peer review, but rather the judgment of student editors to make acceptance decisions) and faculty-edited journals (that tend to rely on peer review), we chose 150 articles from each. We classified articles as empirical if they included original analyses using descriptive or inferential statistics of original or pre-existing quantitative data (e.g., survey studies, content analyses of judicial decisions, meta-analyses).

As described below, we coded features generally related to transparency, such as accessibility, statements about the availability of data, analytic scripts, and other research materials, whether the study was preregistered, and declarations of conflicts of interest and funding sources. We also coded general methodological aspects of those studies, such as whether they were experiments and the types of statistics performed. These provide some background understanding of our sample and may bear on the importance of transparency (e.g., providing analytic code is most relevant to studies using inferential statistics). This is the first study of its kind in empirical legal research, and we are not testing hypotheses; thus, the results should be considered descriptive and exploratory. This study is preregistered and provides open data, code, and materials.

We deviated from previous studies measuring transparency in two main ways. First, previous studies using this type of protocol focused on fields whose journals contain a high proportion of empirical research (e.g., psychology, organizational behavior research, otolaryngology, addiction medicine), ^41^ so they randomly sampled studies without screening out studies that did not use empirical methods. This approach would have been inappropriate for the current study because it would have led us to include a large number of non-empirical studies (~90% of published work, according to a prior estimate). ^42^ As a result, we developed an approach for early screening of non-empirical research (see literature search string below). We also deviated from some previous studies by sampling only from highly ranked journals. This may have biased our results towards finding higher research transparency than the field generally has, because higher rank typically translates to greater selectivity, and thus should in principle enable higher standards. Note also that given the perceived importance of the journals in our sample, low levels of transparency would be especially concerning.

### Identifying empirical articles: Search string used to generate sample

To develop a search string to more efficiently identify and sample articles that met our specifications, we conducted a preliminary examination of the literature. We coded 2019-2020 articles from 10 law journals that Washington and Lee ranks in the top 25 (1,024 total articles). ^43^ Through reading those articles, we identified 92 (or 9% of the sample) meeting our definition of empirical within this dataset. ^44^

Using the knowledge from that preliminary examination, we first considered two different ways of more quickly identifying empirical articles without reviewing the full text. First, we considered selecting only articles with the word “empirical” in the title as Epstein and King had done in their landmark study. However, only 10% of the empirical articles in the preliminary examination sample had the word “empirical” in their title. This strategy, therefore, would miss a great deal of empirical work, raising concerns about the representativeness of the sample and making it more difficult to find our target of 300 recent empirical studies. We also considered selecting only articles with “empirical” in their abstract; however, that strategy would have missed approximately 50% of the articles identified by the more intensive method used in our preliminary examination.

Ultimately, we decided to use the words in the abstracts of the 92 empirical articles we identified in our preliminary examination, and to write a search string based on those words. That search string is:

ABS (“content analysis” OR data* OR behavioral OR behavioural OR empirical OR experiment OR meta-ana* OR multidimensional OR multivariate OR quantitative OR statistical OR study OR studies OR survey OR systematic)

One limitation of this strategy is that, in our preliminary examination, about 8% of the empirical articles we identified did not have an abstract. As a result, any search strategy that uses abstract searches is bound to miss a small proportion of empirical articles, such as commentaries with a trivial empirical component. This may bias our findings towards including more instances of systematic data analysis that would be adverted to in an abstract. Despite this limitation, the search method is efficient (i.e., full text searches would have yielded too many false positives for our team to review) and reproducible (i.e., the full search string and results are provided, as are all exclusions and reasons for exclusion).

Sample

**Figure 1.**
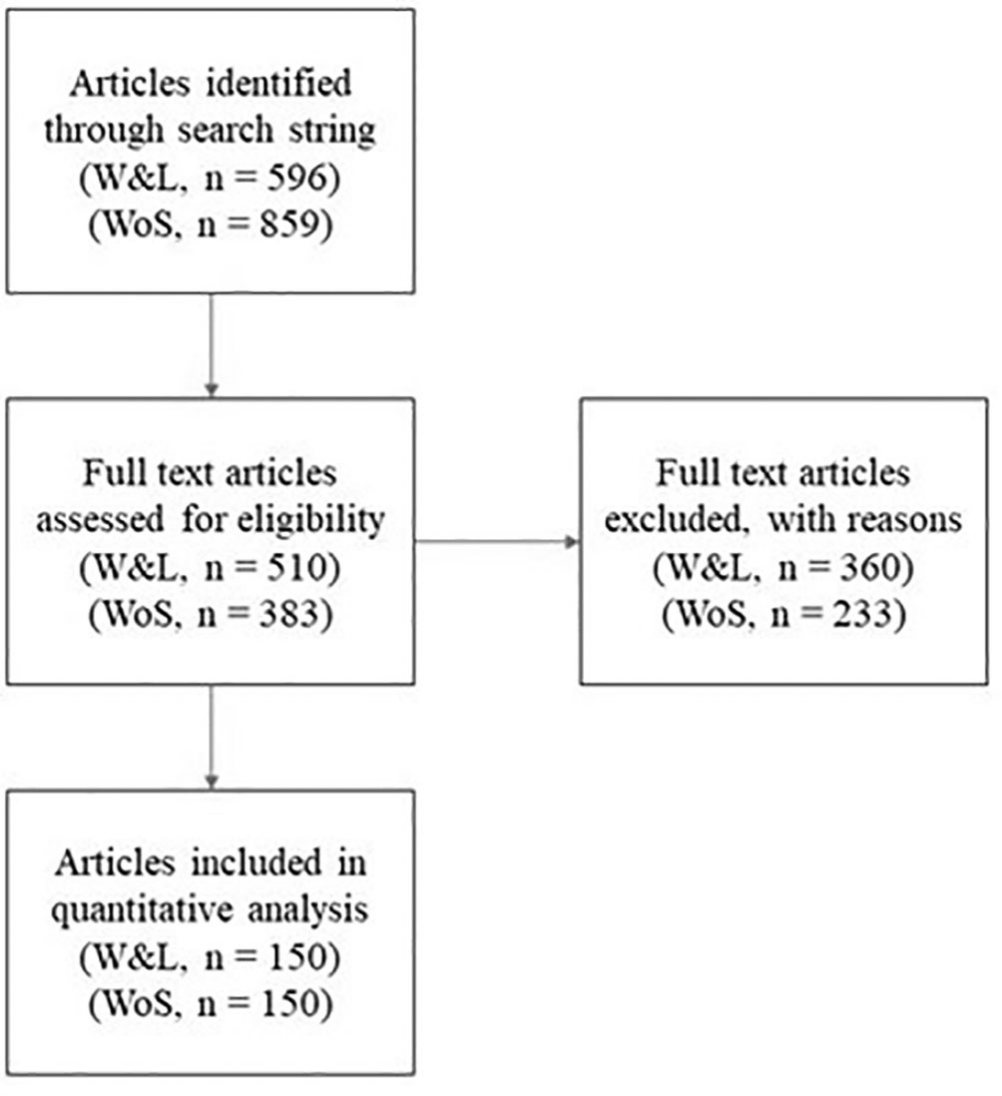
The screening procedure for building the student-edited (W&L) and faculty-edited samples (WoS). Articles were first identified through the Scopus search string described in the methods. They were then screened for eligibility in random order until the samples were complete. The excluded articles and the reasons for their exclusion are available in the E *xtended data*, “W&L screened out” and "Web of Science screened out”.

Figure 1 details our sampling process and exclusions. We used the search string described above to search Scopus for articles published between 1 ^st^ January 2018, and the date of our search, 29 ^th^ January 2021. We populated our overall sample of 300 articles with 150 articles from the top 25 student-edited journals from the Washington and Lee rankings (W&L) (based on its “combined score” in 2019) and 150 articles from the top 25 faculty-edited journals (by 2019 impact factor) in the Web of Science’s “law” database. ^45^ That is, we applied our search string to both of those journal lists. The Washington and Lee search returned 596 articles and the Web of Science search returned 859 articles (see *Extended data*). We decided to sample from high impact journals because we judged that these articles would be most influential among both researchers and policymakers, and thus transparency is especially important.

Because searches returned several of what we classified as non-empirical articles (e.g., the abstract contained the word “data” to describe data regulation laws), one author (JC) randomly sorted both lists and then screened out articles that did not meet our inclusion criterion (i.e., the study includes an analysis of quantitative data) until we reached the pre-specified sample of 150 articles for each group ( Figure 1). Of the 596 articles in the W&L sample, we needed to review 510 to obtain our sample of 150 (i.e., 31% of those reviewed were selected, the rest were excluded). For the Web of Science sample, we needed to review 383 to find 150 empirical articles (i.e., 40.1% of those reviewed were selected, the rest were excluded).

The relatively high rate of exclusions suggests that our search string was overly inclusive, adding more work for us but reducing the chance that we missed a large proportion of empirical articles. The articles screened out and the reasons for their exclusion are described in our *Extended data* (“W&L screened out” and “Web of Science screened out”). After we initiated coding of these articles with the protocol below, we found that 8 were incorrectly categorized as empirical, so we selected the next 8 from the list as replacements. These are the numbers that are reflected in Figure 1 and above.

### Coding procedure

Articles were coded using the structured form developed by Hardwicke and colleagues. ^46^ Following the Hardwicke *et al*., protocol (as well as other transparency coding projects for systematic reviews, see O’Dea *et al*.), ^47^ each article was coded by two of the authors, with disagreements resolved through discussion between those coders and a third author if the coders could not agree (see *Extended data*). The coders were all trained on five articles and did not begin coding the target sample of articles until they reached consensus on the five training articles. As we discuss below, two items were difficult to code, and so we discontinued coding them and do not present the result for them. For multiple-study articles (we defined studies as distinct data collection activities), we coded only the first-reported study. Coding one article in the student-edited sample took about 30-45 minutes. Coding an article in the faculty-edited sample took about 10-20 minutes. This reflects the longer length of the articles in the student-edited sample and that their methods and data were frequently difficult to locate due to the lack of a standard article format. We coded articles from February to September 2021.

The features of the articles that we coded are detailed in the coding sheet and in Table 1 (and further detailed in our preregistration). Some of these features are relevant background information on the studies, such as the statistics used by the researchers, the nature of the data, and data sources. Others are relevant to the transparency and credibility of the research, such as whether authors stated that data and analysis scripts were available, whether the study was preregistered, and whether it was a replication (replications have helped uncover spurious results in prior studies).

**Table 1.** The primary measured variables in our analysis. The full set of variables can be found in the full structured coding form.

Variable	Further details
Article accessibility	• Was the article available through the journal’s website (without university library access, i.e., gold open access)?
	• Was the article available through another service (e.g., ResearchGate, SSRN)?
Conflict of interest	Does the article include a statement indicating whether there were any conflicts of interest?
Funding	Does the article include a statement indicating whether there were funding sources?
Experimental design	Is it an experiment? For our purposes, experiments are studies in which some variable is manipulated by the researcher (e.g., some participants are randomly assigned to a condition).
Synthesis	Is it a synthesis (e.g., meta-analysis, systematic review)? For our purposes, a synthesis is a quantitative analysis of other studies/articles.
Replication	Does the article claim to report a replication study?
Human subjects	Were there human subjects? For our purposes, this means measuring and/or aggregating responses from individuals or groups. This does not include judicial decisions written by judges and analogous data.
Original or secondary data	For our purposes, original data are data the authors collected or generated that did not exist before. Secondary data are data that already existed (e.g., analyses of judicial decisions or contracts).
Data availability	• Does the article state whether or not data are available?
	• How does the statement indicate the data are available?
	• Can you access, download, and open the data files (without contacting the author)?
Analysis script availability	• Does the article state whether or not analysis scripts are available?
	• How does the statement indicate the analysis scripts are available?
	• Can you access, download, and open the analysis files (without contacting the author)?
Materials availability	• Does the article state whether or not materials are available?
	• How does the statement indicate the materials are available?
	• Can you access, download, and open the materials files (without contacting the author)?
Preregistration	• Does the article state whether or not the study (or some aspect of the study) was preregistered?
	• Where does the article indicate the preregistration is located?
	• Can you access and open the preregistration?

With respect to data availability, Hardwicke *et al*. attempted to code whether authors provided a clear reference to where the data could be found (“source of data provided but no explicit availability statement”). ^48^ Due to difficulty coding this item, they did not report this and instead collapsed these types of data references into “no – there was no data availability statement”. Because we expected the current study to include several cases of authors analyzing pre-existing data and datasets, we initially attempted to preserve this as a distinct item in our coding form. However, our coders also encountered difficulty with it (e.g., sometimes articles would provide a vague reference to another article, and, when we accessed that article, it referenced yet other articles). So, our results also collapse these types of data references into the “no data availability statements” category (as we note below, our data availability results are closely in line with Matthews and Rantanen, lending confidence in our data availability conclusions). We did, however, include a separate item for secondary data studies ( Table 1) in which we coded whether authors provided an index of the secondary data items (e.g., references to the judicial decisions included). ^49^

We report 95% confidence intervals calculated using the Sison-Glaz method for multinomial proportions. ^50^

### Deviations from preregistration

Our study deviated from our preregistration in two ways. First, we originally planned to code sample size but did not complete this coding because studies did not provide a single sample size. Second, as noted above, we originally planned to code whether the authors provided the source of the data, but we did not complete this because it was impractical for reasons noted in the previous paragraph.

## Results

Overall, we found a low level of transparency on the characteristics we measured. Only 19% of articles stated that their data are available, and we were able to access that data in only about half of those cases. ^51^ Preregistration and availability of analytic scripts were also very uncommon, and, in fact, almost nonexistent in the empirical legal research examined here. However, we found several positive aspects of the literature to build on. For instance, about 50% of studies employing original data stated that at least some materials were available. In addition, article accessibility was high among the empirical legal research examined here, especially among articles in student-edited journals (100% of those articles were available without library access). These findings are detailed below.

### Sample characteristics

General characteristics of our sample are reported in Table 2, specifically the proportion of articles that: analyzed original or secondary data; used human participants; reported an experiment; were a synthesis (which we operationalized as studies that self-identified as a systematic review or meta-analysis); and reported descriptive or descriptive and inferential statistics. Secondary data analysis was more common (65% of studies, 95% CI = [59%, 70%]) than analysis of original data. Secondary data were also more frequently employed in the student-edited journals (79%, 95% CI = [73%, 85%]) than in the faculty-edited journals (51%, 95% CI = [43%, 59%]). Furthermore, 40% (95% CI = [35%, 46%]) of studies relied on human participants. This figure was 21% (95% CI = [15%, 27%]) among the student-edited journals and 60% (95% CI = [53%, 69%]) among the faculty-edited journals.

**Table 2.** Overview of the samples of empirical legal studies. The variables are: original or secondary data, whether there were human subjects, whether the study was an experiment, whether it was a synthesis (i.e., systematic review or meta-analysis), and whether it used descriptive statistics or descriptive statistics along with inferential statistics. Studies per year can be found in the markdown file in the online data supplement.

Variable	Response	N	% [95% CI]
**All**			
Original or secondary data	Original	106	35% [30%, 41%]
	Secondary	194	65% [59%, 70%]
Human subjects	No	179	60% [54%, 65%]
	Yes	121	40% [35%, 46%]
Experimental design?	No	247	82% [78%, 87%]
	Yes	53	18% [14%, 22%]
Synthesis	No	294	98% [97%, 99%]
	Yes	6	2% [1%, 3%]
Statistics used	Descriptive	97	32% [27%, 38%]
	Descriptive & Inferential	203	68% [62%, 73%]
**Studies in student-edited journals**			
Original or secondary data	Original	32	21% [15%, 28%]
	Secondary	118	79% [73%, 85%]
Human subjects	No	119	79% [73%, 86%]
	Yes	31	21% [15%, 27%]
Experimental design?	No	129	86% [81%, 92%]
	Yes	21	14% [9%, 20%]
Synthesis	No	150	100% [100%, 100%]
	Yes	0	0% [0%, 1%]
Statistics used	Descriptive	64	43% [35%, 51%]
	Descriptive & Inferential	86	57% [49%, 65%]
**Studies in faculty-edited journals**			
Original or secondary data	Original	74	49% [41%, 58%]
	Secondary	76	51% [43%, 59%]
Human subjects	No	60	40% [33%, 49%]
	Yes	90	60% [53%, 69%]
Experimental design?	No	118	79% [73%, 85%]
	Yes	32	21% [15%, 28%]
Synthesis	No	144	96% [93%, 99%]
	Yes	6	4% [1%, 7%]
Statistics used	Descriptive	33	22% [16%, 29%]
	Descriptive & Inferential	117	78% [72%, 85%]

Turning to methodology, our sample contained fewer experiments (which require random assignment according to our definition) relative to secondary data analyses (18% of studies, 95% CI = [14%, 22%]). Syntheses were very uncommon, with only six in the sample (all six in the faculty-edited sample). Most articles (68% (95% CI = [62%, 73%])) contained descriptive *and* inferential statistics (the remaining 32% reported only descriptive statistics). 78% (95% CI = [72%, 85%]) of the faculty-edited articles used inferential statistics versus 57% (95% CI = [49%, 65%]) in the student-edited sample.

Among the 194 articles that used secondary data, 53 or 27% (95% CI = [21%, 35%]) of articles analyzed judicial decisions, 11 (6% (95% CI = [0%, 13%])) analyzed company documents, and a further 11 analyzed statutes or legislation (see “table 2secondary” in *Extended data*). Human participants were recruited from a variety of groups, with 12 of the 121 articles (10% (95% CI = [2%, 19%])) sampling from university students, 35 (29% (95% CI = [21%, 38%])) sampling from the general population, and 74 (61% (95% CI = [53%, 70%])) sampling from special populations. Those special populations ^52^ included difficult-to-reach groups such as judges, young offenders, and government employees (see “table 2 special” in *Extended data*).

### Article accessibility

**Figure 2.**
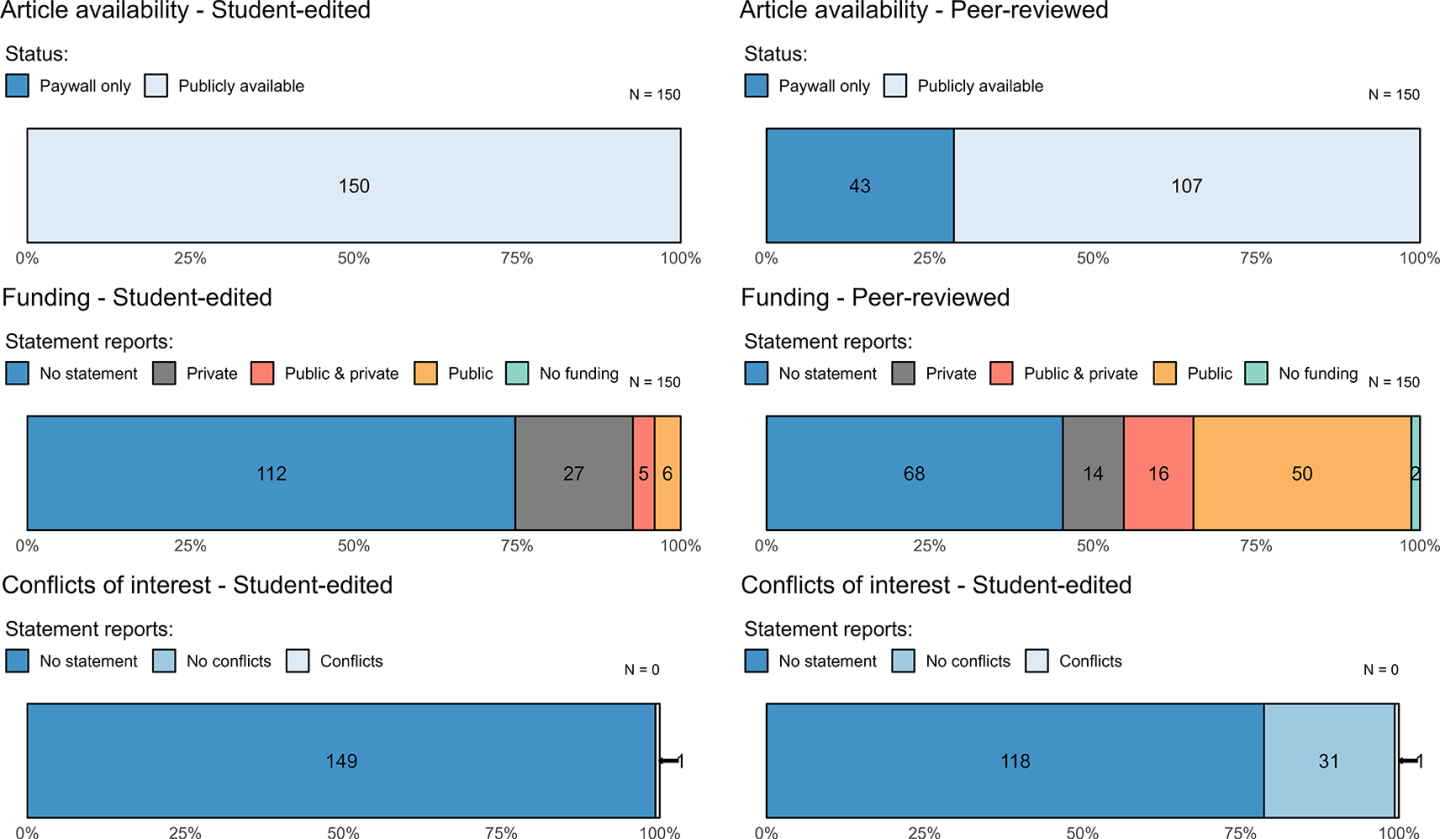
Article availability, funding statements, and conflict of interest statements in empirical legal research. The left column includes articles from the student-edited sample and the right column is from the faculty-edited sample. Numbers within bars refer to the number of articles that meet the given standard.

The articles in our sample were generally easy to access as compared to estimates from previous metascientific studies in criminology and psychology ( Table 3, Figure 2). ^53^ 86% (95% CI = [82%, 90%]) of articles had publicly available versions – 100% of the student-edited journal articles and 71% (95% CI = [65%, 79%]) of the faculty-edited group. 70% of articles (95% CI = [65%, 76%]) were gold open access, meaning they were accessible on journals’ websites. This was the case for 100% of the articles in student-edited journals, whereas 41% (95% CI = [33%, 49%]) of the faculty-edited articles were gold open access. Empirical legal researchers also regularly use pre- and post-print services to provide open access versions of their work. 42% (95% CI = [36%, 48%]) of articles in the overall sample were downloadable on SSRN and 22% (95% CI = [18%, 27%]) were downloadable on ResearchGate.

**Table 3.** Transparency and credibility-related features of empirical legal research. The variables are: article accessibility, the presence and content (if applicable) of statements about funding, conflicts of interest, data availability, materials availability, and analysis script availability. We further coded whether there was a statement that the study was preregistered and whether the authors described the study as a replication. The figures for materials availability include only the articles that collected original data. Note that this figure reflects availability statements. As discussed in text, actual accessibility was considerably lower.

	All			Student-edited		Faculty-edited	
Variable	Response	N	% [95% CI]	N	% [95% CI]	N	% [95% CI]
Article accessibility	Paywall only	43	14% [11%, 18%]	0	0% [0%, 1%]	43	29% [22%, 36%]
	Available	257	86% [82%, 90%]	150	100% [100%, 100%]	107	71% [65%, 79%]
Conflicts of interest	No statement	267	89% [86%, 93%]	149	99% [99%, 100%]	118	79% [73%, 85%]
	Conflicts	2	1% [0%, 4%]	1	1% [0%, 2%]	1	1% [0%, 7%]
	No conflicts	31	10% [7%, 14%]	0	0% [0%, 1%]	31	21% [15%, 27%]
Funding	No statement	180	60% [55%, 66%]	112	75% [69%, 82%]	68	45% [37%, 54%]
	No funding	2	1% [0%, 6%]	0	0% [0%, 7%]	2	1% [0%, 10%]
	Private	41	14% [8%, 19%]	27	18% [12%, 25%]	14	9% [1%, 18%]
	Public	56	19% [13%, 24%]	6	4% [0%, 11%]	50	33% [25%, 42%]
	Public & private	21	7% [2%, 13%]	5	3% [0%, 11%]	16	11% [3%, 19%]
Data availability	No statement	242	81% [76%, 85%]	124	83% [77%, 89%]	118	79% [73%, 85%]
	Says available	57	19% [15%, 23%]	25	17% [11%, 23%]	32	21% [15%, 28%]
	Not available	1	0% [0%, 5%]	1	1% [0%, 7%]	0	0% [0%, 7%]
Analysis script availability	No statement	281	94% [91%, 96%]	142	94% [91%, 96%]	139	93% [89%, 97%]
	Says available	19	6% [4%, 9%]	8	6% [4%, 9%]	11	7% [4%, 12%]
Materials availability	No statement	59	56% [46%, 65%]	17	53% [38%, 71%]	42	57% [46%, 68%]
	Says available	47	44% [35%, 54%]	15	47% [31%, 65%]	32	43% [32%, 55%]
Preregistration	No statement	292	97% [96%, 99%]	147	98% [97%, 100%]	145	97% [95%, 100%]
	Says preregistered	8	3% [1%, 5%]	3	2% [1%, 4%]	5	3% [1%, 6%]
Replication	No	289	96% [95%, 98%]	147	98% [97%, 100%]	142	95% [92%, 98%]
	Yes	11	4% [2%, 6%]	3	2% [1%, 4%]	8	5% [3%, 9%]

### Conflicts of interest and funding statements

Turning to conflicts of interest and funding statements, we found that most articles did not provide any such declaration. In fact, only 11% (95% CI = [8%, 15%]) of articles include a conflicts of interest statement. Conflicts of interest statements were more common in the faculty-edited journals with only one article in the student-edited sample containing such a statement. As to statements of funding sources, 40% (95% CI = [35%, 46%]) of articles contained a statement. Again, such statements appear to be rarer in the student-edited sample (see Table 3).

### Data availability

**Figure 3.**
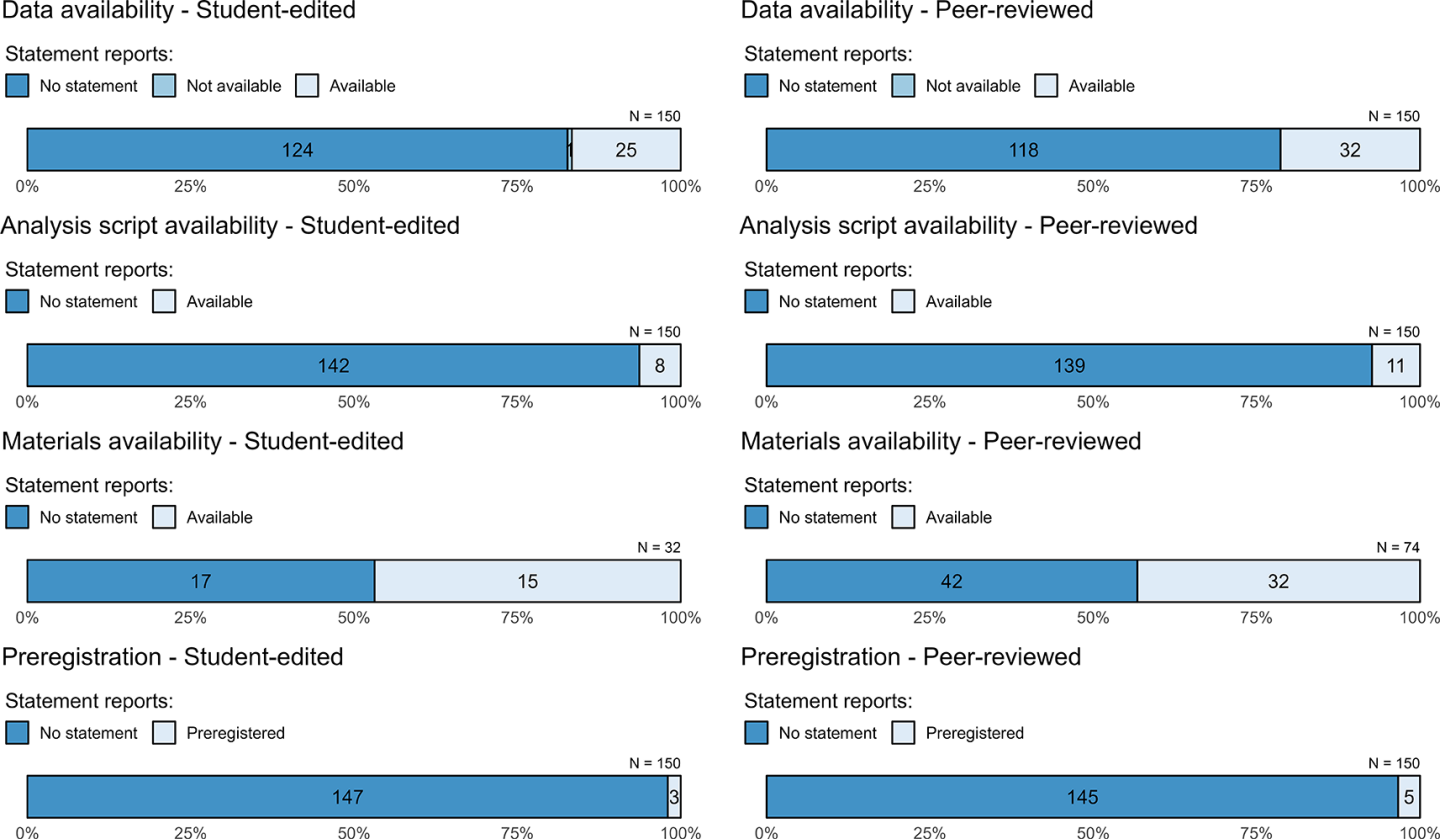
Assessment of transparency and credibility-related characteristics of empirical legal research. The student-edited sample is reported in the left column and the right column is the faculty-edited sample. Numbers within bars refer to the number of articles that meet the given standard. Data availability, analysis script availability, and preregistration bars include the full sample (150 per group), whereas the bars for materials availability include only the articles that collected original data. Note that this figure reflects availability statements, whereas, discussed in text, actual accessibility was considerably lower.

The availability of the data, analysis scripts, and materials in our sample was generally low ( Table 3, Figure 3). Just 19% (95% CI = [15%, 23%]) of articles provided a statement that data are available. Of articles with data availability statements, the most common means for sharing data were via a third-party repository (39%, 95% CI = [26%, 53%]), by contacting the author (28%, 95% CI = [16%, 42%], and via a personal or institutional website (21%, 95% CI = [9%, 35%]) (see “tabledatahow” in *Extended data*). We checked whether the data referenced in the statements were readily available (i.e., whether we could access them without further steps, such as contacting the author). Only about half (53%, 95% CI = [40%, 66%]) were readily available, making the effective data availability rate about 10%. This figure closely corresponds to Matthews and Rantanen’s 12% estimate of data availability (also without contacting authors) at predominantly student-edited journals published from 2010 to 2019. ^54^

In the social sciences, much of the move towards providing data availability statements has occurred in the context of psychological research, where original data are often collected. As a result, it may be useful to drill down on articles reporting on original data. Limiting our analysis to these articles (N = 106), we found 29% (95% CI = [22%, 39%]) included a data availability statement, whereas only 13% (95% CI = [9%, 18%]) of articles reporting on secondary data did so (N = 194).

For secondary data, as noted above, we coded the steps authors took to provide information about the dataset. In most cases, authors did not provide any details about the dataset (see “table_secondarySteps” in *Extended data*). In 26 of the 194 (13%, 95% CI = [8%, 20%]) articles reporting on secondary data, the authors provided an index of the secondary data (e.g., a list of judicial decisions relied on). Several others linked to sources, such as external websites, that were no longer accessible.

### Analysis-script availability

Very few studies included a statement about the availability of their analysis scripts (6%, 95% CI = [4%, 9%]). Providing analysis code is especially important when reporting inferential statistics (e.g., to determine the exact statistical test and assumptions the authors used), but of the 203 studies that relied on inferential statistics, only 8% (95% CI = [5%, 12%]) made their code available. Even these figures are somewhat inflated, however, because only for approximately half of the articles with script availability statements could we access the scripts without taking further steps (again, due to dead links and statements indicating that the scripts were available on request).

### Materials availability

The materials availability results presented in Table 3 and Figure 3 are limited to studies with original data. We presented them this way because sharing of study materials (e.g., survey instruments, vignettes) is arguably less applicable to analysis of existing data. However, some studies analyzing secondary data do involve useful materials that could be shared, such as the coding sheets used by researchers who tally different sorts of judicial decisions. Of studies that reported on original data, about 44% (95% CI = [35%, 54%]) stated that materials were available. Recall that this figure does not mean that all materials were made available, but rather that authors stated that at least some materials were available. Moreover, we were able to access materials for only 39 of the 47 (83%, 95% CI = [74%, 94%]) studies that stated that materials were available, making the effective material available rate about 37% among studies that report on original data.

### Preregistration

Almost no studies reported being preregistered (3%, 95% CI = [1%, 5%]). Of the 8 preregistered studies, we could not access the preregistrations of 2. The purported locations of the 8 preregistrations were: the Open Science Framework (5 studies), the AsPredicted.org registry (1 study), the PROSPERO registry (for syntheses; 1 study), and the Evidence in Governance and Politics (EGAP) registry (which is hosted by the Open Science Framework; 1 study).

### Replication

Very few studies stated that they were reporting the results of a replication (4%, 95% CI = [2%, 6%]).

## Discussion

Our results suggest that there is ample room to improve empirical legal research transparency. Our hope is that our results encourage researchers in the field of quantitative empirical legal research to move forward in making their work verifiable and reusable. Articles in our sample generally had low levels of transparency and credibility-related characteristics that we measured. These results are not much different than many other fields, as shown in Table 4. ^55^ We identified the studies in Table 4 non-systematically, based on studies we were aware from an informal literature search.

On a more positive note, with respect to article accessibility, empirical legal research performs very well, especially for articles published in student-edited journals. Of course, accessibility without fuller transparency risks readers relying on unverifiable results. Ideally, research should be fully transparent and accessible.

**Table 4.** A comparison of studies measuring transparency-related factors.

		Current study	Hardwicke *et al*., 2020	Johnson *et al*., 2020	Culina *et al*., 2020	Hardwicke *et al*., 2018a
Field(s)		Empirical Legal	Social Sciences	Otolaryngology	Ecology	Psychology
Reform(s)?		--	--	--	Journal guidelines	Journal guidelines
Articles analyzed	*N*	300	250	300	346	174
	Publication years	2018-2021	2014-2017	2014-2018	2015-2019	2015-2017
Article availability	Paywall only	14%	54.0%	77.7%	--	--
	Publicly available	86%	40.4%	22.3%	--	--
Data availability	No statement	81%	80.8%	96.7%	--	22%
	Says available	19%	7.0%	2.0%	79% *	78%
	Not available	0%	0.6%	1.3%	--	0%
	*N*	300	156	151	346	174
Analysis script availability	No statement	94%	98.7%	99.4%	--	--
	Says available	6%	1.3%	0.7%	27%	--
	*N*	300	156	151	346	--
Materials availability	No statement	56%	89.4%	94.5%	--	--
	Says available	44%	10.6%	4.8%	--	--
	*N*	106	151	145	--	--
Preregistration	No statement	97%	100%	95.4%	--	--
	Says preregistered	3%	0%	4.0%	--	--
	*N*	300	156	151	--	--
Replication	No	96%	98.7%	100%	--	--
	Yes	4%	1.3%	0%	--	--
	*N*	300	156	151	--	--

Culina *et al*. (2020) and Hardwicke *et al*. (2018a) focused on journals that had recently implemented transparency guidelines (Culina *et al*. studied such journals in Ecology; Hardwicke *et al*. focused on the journal, *Cognition*).

* Culina *et al*. did not study availability statements, but data availability *per se* – a fuller description of the methodological differences between these studies and an expanded table is available ( *Extended data*, “Table 4 - online supplement”).

Comparing student-edited and faculty-edited journals on other transparency and credibility-related characteristics, we generally did not find large differences. However, student-edited journals did seem to have a smaller proportion of articles with conflicts of interest and funding statements. Deficiencies in reporting funding may be due to law professors relying largely on internal funding that they do not see as important to report. While such funding might raise fewer concerns than that from external sources, it is impossible for the reader to know – without a statement – whether a study received funding and from what source. The best practice, one we saw among some articles in our sample, would be to *explicitly* declare funding sources and conflicts or the lack thereof, and law journals should require these declarations. Moreover, many legal researchers may have affiliations that should be disclosed, such as governmental appointments, affiliations with think tanks, and company directorships or board memberships.

While we urge caution in comparing our results to those from transparency studies of other fields, such a comparison may be instructive in some ways (see Table 4). In particular, we did not observe large differences (other than in materials availability, see below) between empirical legal research and other fields. However, the two comparison studies in Table 4 (sampling from social science generally and otolaryngology) did not restrict their samples based on journal ranking, ^56^ whereas our study sampled only from what many would describe as the top journals in the field. It arguably would be reasonable to expect that these journals should be leading the field in producing verifiable and reusable work. Moreover, the other studies focus on articles published in the mid-2010s, and so we might expect stronger adoption of transparency and credibility reforms in our sample. In other words, the results of our study likely provide an optimistic comparison with other fields of research.

Regarding the effects of reforms, Table 4 also contains two comparisons with studies that have sampled only from journals that have implemented transparency and openness guidelines. In particular, Culina and colleagues sampled only from ecology journals that had implemented data and analysis script availability policies (both mandatory guidelines and encouragements). ^57^ In addition, Hardwicke *et al*., examined data availability of studies published by the journal *Cognition*, which had implemented a mandatory data availability policy. ^58^ As can be seen in Table 4, recent articles in those journals show markedly higher levels of data and script availability than our study found in empirical legal research. We cannot say what caused the relatively high levels of data and script availability in these journals, but these results suggest journal guidelines may play an important role in reform efforts. However, seeing as Matthews and Rantanen found that the *Journal of Empirical Legal Studies* underperformed student-edited law journals despite having a policy that encourages data sharing, it seems unlikely that mere encouragements are sufficient.

Our results might be limited in other respects. First, empirical legal research is a multi-disciplinary field, which uses a panoply of methods from several research traditions. ^59^ As a result, some forms of transparency may be less applicable for some methods than for others. We attempted to take this into account by reporting results for some of these practices separately for different types of studies (e.g., reporting materials transparency for studies reporting on original data; reporting analysis script transparency for studies reporting inferential statistics). In this respect, our results may overestimate transparency levels by restricting analyses to only one subset of studies, when in fact the practice would be beneficial for a broader range of studies. For example, many studies reporting on secondary data would nevertheless be more reproducible if they shared materials such as coding sheets used by research assistants who coded legislation or judicial decisions. ^60^

Second, we did not contact authors to determine whether statements that data, materials, or analysis scripts were available upon request would be honored or whether authors of studies that do not mention availability would disclose information upon request. As noted above, however, multiple studies have found that most authors do not provide their data when requested, even when their paper includes a statement indicating that data are available upon request. ^61^ Most recently, Gabelica and colleagues found that authors provided just 7% of 1,792 requested datasets despite the authors indicating that the data were available. ^62^ While some authors may have responded to our requests, relying on author responses is problematic in the long run because researchers retire or otherwise leave academia, leading to a “rapid” decrease of research data availability over time. ^63^ In addition, this method of transparency presents a significant obstacle for third parties who wish to access these artifacts for purposes that the authors may view as not in the authors’ interests (e.g., because the requesters suspect an error in the original article). The importance of posting data, as opposed to promising to make it available upon request, has been recognized by government funders, some of whom require authors of funded studies to post data upon publication. ^64^

Third, we did not attempt to take into account data sharing limits such as privacy and proprietary datasets. ^65^ However, we did code whether any statement was made about data availability, which would have included statements about barriers to sharing data, and we did not find any studies that explained their lack of data sharing in such terms, so this may not have been prevalent. Alternatively, authors simply might not have reported their inability to share the data. Moreover, we attempted to code other means of transparency for secondary data analysis (e.g., indexes of cases relied on) and found that few papers took up any such options. Future metaresearch projects may wish to take a more focused approach, targeting specific empirical legal research methods to better understand their norms and limits related to transparent research and reporting. ^66^

Fourth, our coding is only current as of September 2021. If, for example, articles have since been edited to indicate data availability, our results will not reflect that. While that is unlikely, it is perhaps more probable that some articles were temporarily open access because they had just been released, but have now moved behind paywalls. As a result, our results may overestimate open access, especially among the faculty-edited journals published by commercial publishers.

Fifth, using the impact factor metric for Web of Science to identify faculty-edited law journals may have included journals that some in the empirical legal research community would disagree about as important journals in the field. For instance, the impact factor for the *Journal of Empirical Legal Studies* resulted in it not being included, despite it being the journal produced by one of the main societies in the field. However, including journals based on our subjective judgment would have introduced bias into the findings. And, our results for data availability closely matched that of Matthews and Rantanen, who did study the *Journal of Empirical Legal Studies.*

Sixth, our sample is potentially biased. If the studies we initially found to develop our search string are different in important ways from the population of studies, the generalizability of our results is limited. That said, our initial sample is sizeable. It includes nearly 100 studies, which reduces the likelihood that we missed sets of relevant studies that are either more or less transparent than the studies in our sample. The bias, of course, depends on the variability of terms in the population of abstracts. In our view, however, the search string terms fairly represent common empirical legal methods and words used to describe them in the literature (e.g., content analysis, behavioral). This gives us confidence that our results describe, at a minimum, a relevant portion of the empirical legal studies literature.

We also highlight that the mere presence of data, analysis scripts, and preregistration does not mean that associated findings will be reproducible. Systematic research has found that data is often not well documented, making it difficult to reproduce findings. ^67^ Future projects should consider focusing on a smaller number of studies for which some data are available to determine if the results are fully reproducible. ^68^ Similarly, other aspects of research quality, such as whether preregistrations were actually followed, are an important avenue for future research.

**Looking forward**

Where do we go from here? As we reviewed above, transparency has proven vital in uncovering flaws, limitations, and fraud in published work. We call on journals to adopt policies to increase the transparency of published studiessuch as open data and code. ^69^ Such policies can be augmented by “verification checks” whereby the journal verifies all disclosures and uses the disclosed data and code to verify that the article’s results are reproducible. The American Economic Association, for example, performs third-party verifications on all articles published in its journals. ^70^ This may be especially important for journals that are not commonly peer reviewed, such as student-edited journals, because peer review detects some flaws and errors. ^71^ Even then, however, studies have found that peer reviewers detect just a minority of errors deliberately added to the reviewed studies. ^72^ Only with a high level of transparency can we hope that errors in important studies are likely to be caught, as transparency enables robust post-publication peer review.

The fact that at least some datasets employed in empirical legal research studies are proprietary and cannot be made publicly available should not cause the field to shy away from general data availability requirements. For example, in psychology it is common for privacy issues to preclude data sharing. Journal guidelines in this field sometimes balance privacy and other ethical constraints on data sharing with data availability by asking authors to explain any restrictions in the manuscript and requiring data sharing if such an explanation cannot be provided. ^73^ An example of such a statement is: “The conditions of our ethics approval do not permit public archiving of anonymized study data. Readers seeking access to the data should contact the lead author X or the local ethics committee at the Department of Y, University of Z. Access will be granted to named individuals in accordance with ethical procedures governing the reuse of sensitive data. Specifically, requestors must meet the following conditions to obtain the data [insert any conditions, e.g., completion of a formal data sharing agreement, or state explicitly if there are no conditions].” ^74^ This policy is consistent with TOP guidelines for data transparency (Level II), which require data to be posted to a trusted repository and any exceptions to be explained in the article. ^75^ Editors might also consider requiring authors who use proprietary data to include explicit statements related to limitations that arise from the inability to verify claims derived from such data. Specifically, readers should be explicitly warned about relying on unverifiable results.

Ideally, incentive structures for researchers should reward transparency and reproducibility. This includes the research assessment involved in hiring and promotions. ^76^ Research funders should also promote transparency by making it a requirement of funding in appropriate cases. In promising steps, the U.S. President and his administration declared 2023 the Year of Open Science, ^77^ and the U.S. National Institutes of Health ^78^ and the U.S. Department of Education ^79^ both recently instituted data sharing policies for research they fund.

Finally, empirical legal research can take advantage of the larger movement in the social sciences, medicine, and many other fields, by leveraging the technology, training, and ideas flowing from those credibility revolutions. Free technologies like the Open Science Framework provide a place not just to store data, but to collaborate, establish version control, preregister, and store video stimuli. Other examples include tools like Github (a data and code repository), AsPredicted (a general study registry), Declare Design (a tool for creating a preregistration), and the American Economic Association’s registry for randomized controlled trials. Straightforward guides to data staring, preregistering, and many other transparency and credibility-related activities are now available. ^80^ At least one guide specific to some empirical legal research methodologies is also available, and we hope more are on the way. ^81^ With these tools at their fingertips – and as a field whose data and results are often of great public importance – there is little reason researchers in the field of empirical legal research should not become leaders in the move towards transparency and credibility.

## Data Availability

OSF: Transparency and reproducibility-related practices in empirical legal research https://osf.io/msjqf/. This project contains the following underlying data:
• Raw data 1 ( https://osf.io/ktpcd) – original data • Raw data 2 ( https://osf.io/jx7fe) – replacement articles for incorrectly included articles OSF: Transparency and reproducibility-related practices in empirical legal research https://osf.io/msjqf/. This project contains the following extended data:
• W&L screened out ( https://osf.io/qf7sc) – articles from the W&L database that were screened and the reasons for that • Web of Science screened out ( https://osf.io/vbu63) – articles from the W&L database that were screened and the reasons for that • Disagreements ( https://osf.io/7q32m) – articles the coders disagreed on • table 2secondary ( https://osf.io/usfy4) – the types of secondary datasets and their frequencies • table 2special ( https://osf.io/m589c) – the types of special populations surveyed and their frequencies • tabledatahow ( https://osf.io/67t9y) – how datasets were made available and their frequencies • table_secondarySteps ( https://osf.io/xczpy) – steps authors conducting secondary data analyses took to make their data available •
Table 4 - online supplement ( https://osf.io/z6tx3) – methods differences between studies in Table 4

## Footnotes

^1^
Marcus R. Munafò *et al*., *A manifesto for reproducible science*, 1 N at. H um. B ehav. 1 (2017) at 4-5.

^2^

*Id.*; Joshua D. Angrist and Jörn-Steffen Pischke, *The credibility revolution in empirical economics: How better research design is taking the con out of econometrics*, 24(2) J. E con. P erspect. 3 (2010); Simine Vazire, *Implications of the credibility revolution for productivity, creativity, and progress*, 13(4) PERSPECT. PSYCHOL. SCI. 411 (2018); Garret Christensen *et al*., *Open Science Practices are on the Rise: The State of Social Science (3S) Survey*, *MetaArXiv*, https://osf.io/preprints/metaarxiv/5rksu (accessed 2022).

^3^
Munafò *et al*., *supra* note 1 at 2-3.

^4^
C ary F unk *et al*., *Trust and Mistrust in Americans’ Views of Scientific Experts*, Pew Research Center (2019) 24; Justin T. Pickett and Sean Patrick Roche, *Questionable, Objectionable or Criminal? Public Opinion on Data Fraud and Selective Reporting in Science*, 24 S ci. E ng. E thics 151 (2018).

^5^
Kathryn Zeiler, *The Future of Empirical Legal Scholarship: Where Might We Go from Here?* 66 J. L egal E duc. 78 (2016); Jason M. Chin, Malgorzata Lagisz and Shinichi Nakagawa, *Where is the evidence in evidence-based law reform?* 45(3) U.N.S.W.L.J. 1124 (2021); Abigail Matthews and Jason Rantanen, *Legal Research as a Collective Enterprise: An Examination of Data Availability in Empirical Legal Scholarship*, *SSRN*, https://papers.ssrn.com/sol3/papers.cfm?abstract_id=4057663 (accessed 2022) at 4.

^6^
This generally tracks the definition provided in Michael Heise, *The past, present, and future of empirical legal scholarship: judicial decision making and the new empiricism*, U niv. I ll. L aw R ev. 819 (2002); we acknowledge that many definitions of empirical legal research have been offered, see Shari Seidman Diamond and Pam Mueller, *Empirical Legal Scholarship in Law Reviews*, 6 A nnu. R ev. L aw S oc. S ci. 581 (2010) at 582-583. As we detail below, our definition is useful for the present study, which measures transparent practices.

^7^
Vazire, *supra* note 2; Munafò, *supra* note 1.

^8^
Monya Baker, *1,500 scientists lift the lid on reproducibility*, 533 N at. 452 (2016).

^9^
Open Science Collaboration (OSC), *Estimating the Reproducibility of Psychological Science*, 349 S cience 3451 (2015); Richard A. Klein *et al*., *Investigating variation in replicability: A ‘many labs’ replication project*, 45(3) S oc. P sychol. 142 (2014); Richard A. Klein *et al*., Many Labs 2: Investigating Variation in Replicability Across Samples and Settings, 1 A dv. M eth. & P ract. P sychol. S ci. 443 (2018); Charles Ebersole *et al*., *Many Labs 3: Evaluating participant pool quality across the academic semester via replication*, 67 J. E xp. S oc. P sychol. 68 (2016); Richard A. Klein *et al*., *Many Labs 4: Failure to Replicate Mortality Salience Effect With and Without Original Author Involvement*, https://psyarxiv.com/vef2c (accessed 2022); Colin F. Camerer *et al*., *Evaluating replicability of laboratory experiments in economics*, 351 Science 1433 (2016); Colin F. Camerer *et al*., *Evaluating the replicability of social science experiments in Nature and Science between 2010 and 2015*, 2 N at. H um. B ehav. 637 (2018).

^10^
See Leif D. Nelson, Joseph Simmons, Uri Simonsohn, *Psychology’s Renaissance*, 69 A nnu. R ev. P sychol. 511 (2018); Leslie K. John *et al*., *Measuring the Prevalence of Questionable Research Practices With Incentives for Truth Telling*, 23(5) P sychol. S ci. 524 (2012).

^11^
Angrist and Pischke, *supra* note 2; Sarah Necker, *Scientific misbehavior in economics*, 43 R es. P ol. 1747 (2014).

^12^
Jason M. Chin *et al*., Questionable Research Practices and Open Science in Quantitative Criminology, J. Q uant. C rim. (2021).

^13^
See e.g., Garret Christensen and Edward Miguel, *Transparency, Reproducibility, and the Credibility of Economics Research*, 56 J. E con. L it. 920 (2018); Andrew C. Chang and Phillip Li, *Is Economics Research Replicable? Sixty Published Papers from Thirteen Journals Say “Often Not,”* 11 C rit. F in. R ev. 185 (2022) (finding the lack of replicability is due mainly to lack of data availability). In economics, secondary data is referred to as “observational data.”

^14^
Vazire, *supra* note 2; E. Miguel, *et al.*, *Promoting Transparency in Social Science Research*, 343 S cience 30 (2014).

^15^
Iain Chalmers and Paul Glasziou, *Avoidable waste in the production and reporting of research evidence*, 374 L ancet 86 (2009).

^16^
Timothy H. Vines *et al*., *The Availability of Research Data Declines Rapidly with Article Age*, 24 C urr. B iol. 94 (2014); Jelte M. Wicherts *et al*., *Willingness to Share Research Data is Related to the Strength of the Evidence and the Quality of Reporting of Statistical Results*, 6(11) 1 PL oS ONE (2011).

^17^

*Id.*

^18^
Simine Vazire and Alex O. Holcombe, *Where are the Self-Correcting Mechanisms in Science?*, 26(2) R ev. G en. P sychol. (2022).

^19^
Uri Simonsohn, Joseph Simmons and Leif D. Nelson, *[98] Evidence of Fraud in an Influential Field Experiment About Dishonesty*, http://datacolada.org/98 (accessed 2022).

^20^
See: Brigitte C. Madrian, *Applying Insights from Behavioral Economics to Policy Design*, 6 A nnu. R ev. E conom. 663 (2014).

^21^

Cabinet Office Behavioural Insights Team, Applying behavioural insights to reduce fraud, error and debt (2012), https://vng.nl/sites/default/files/knowledge_base_compliance/Rapport_201608_Applying_behavioural_insights.pdf (accessed 2022)

^22^
Francesca Gino, Gino-memo-data-colada-August16.pdf, http://datacolada.org/storage_strong/Gino-memo-data-colada-August16.pdf (accessed 2022) [ **emphasis added**].

^23^
Fountain N, et al.: Fabricated data in research about honesty. You can’t make this stuff up. Or, can you? NPR 2018. https://www.npr.org/2023/07/27/1190568472/dan-ariely-francesca-gino-harvard-dishonesty-fabricated-data.

^24^
Brian A. Nosek *et al*., *The Preregistration Revolution*, 115(11) PNAS 2600 (2018).

^25^
Christopher D. Chambers and Loukia Tzavella, *The past, present and future of Registered Reports*, 6 N at. H um. B ehav. 29 (2021).

^26^
Anne M. Scheel, Mitchell Schijen and Daniël Lakens, *An excess of positive results: Comparing the standard Psychology literature with Registered Reports*, 4(2) AMPPS (2021).

^27^
Tom E. Hardwicke *et al*., *An empirical assessment of transparency and reproducibility-related research practices in the social sciences (2014–2017)*, 7(2) R. S oc. O pen sci. 190806 (2020); Tom E. Hardwicke *et al*., *Estimating the Prevalence of Transparency and Reproducibility-Related Research Practices in Psychology (2014–2017)*, P erspect. P sychol. S ci. (2021); Austin L. Johnson *et al*., *An assessment of transparency and reproducibility-related research practices in otolaryngology*, 130(8) T he L aryngoscope 1894 (2020); Mopileola Tomi Adewumi *et al*., *An evaluation of the practice of transparency and reproducibility in addiction medicine literature*, 112 A ddictive B ehaviors 106560 (2021); Elizabeth R. Tenney *et al*., *Open Science and Reform Practices in Organizational Behavior Research over Time (2011 to 2019)*, https://psyarxiv.com/vr7f9/ (accessed 2022).

^28^

Hardwicke *et al*., 2021, *Id.*

^29^

*Id.*

^30^
Nelson *et al*., *supra* note 10.

^31^
Tom E. Hardwicke *et al*., *Data availability, reusability, and analytic reproducibility: evaluating the impact of a mandatory open data policy at the journal Cognition*, 5 R. S oc. O pen sci. 180448 (2018a); Anisa Rowhani-Farid and Adrian G. Barnett, *Has open data arrived at the British Medical Journal (BMJ)? An observational study*, 6 BMJ O pen e011784 (2016); Antica Culina *et al*., *Low availability of code in ecology: A call for urgent action*, 18(7) PL oS B iol. e3000763 (2020).

^32^
Christensen *et al*., *supra* note 2.

^33^
Lee Epstein and Gary King, *The Rules of Inference*, 69 U. C hi. L. R ev. 1 (2002).

^34^
Shari Seidman Diamond, *Empirical Legal Scholarship: Observations on Moving Forward*, 113 N w. U. L. R ev. 1229 (2019).; Zeiler, *supra* note 5; Gregory Mitchell, *Empirical legal scholarship as scientific dialogue*, 83 N.C. L. R ev. 167 (2004). In other metaresearch in empirical legal research, Diamond and Mueller ( *supra* note 6) tracked the amount of quantitative and qualitative empirical research in law journals, finding that only about 10% of articles in highly ranked U.S. law journals contained original empirical work; see also Michael Heise, *An Empirical Analysis of Empirical Legal Scholarship Production*, 1990-2009, 2011 U. I ll. L. R ev. 1739 (2011). And Hall and Wright examined trends in the use of one particular empirical legal research methodology—systematic analysis of judicial decisions. They found that papers in this area rarely cited methodological articles and seemed to reinvent the wheel, methodologically, in each iteration: Mark A. Hall and Ronald F. Wright, *Systematic Content Analysis of Judicial Opinions*, 96 C alif. L. R ev. 63 (2008).

^35^
Kanu Okike *et al*., *Single-blind vs Double-blind Peer Review in the Setting of Author Prestige*, 316 JAMA 1315 (2016); Huber, Jürgen et al, *Nobel and novice: Author prominence affects peer review*, 119(41) PNAS e2205779119 (2022); Simine Vazire, *Our obsession with eminence warps research*, 547 N at. 7 (2019).

^36^
Huber *et al*, *id.*

^37^
Matthews and Rantanen, *supra* note 5.

^38^
Epstein and King, *supra* note 33 at 15-16.

^39^
N = 250 in Hardwicke *et al*., 2020, *supra* note 27; N = 250 in Hardwicke *et al*., 2021, *supra* note 27; N = 286 in Johnson *et al*., *supra* note 27; N = 244 in Adewumi *et al*., *supra* note 27; N = 2234 in Tenney *et al*., *supra* note 27; N = 300 also provides a margin of sampling error of about 6%, although we did not include this in our *a priori* justification: American Association for Public Opinion Research, Margin of Sampling Error/Credibility Interval, https://www.aapor.org/Education-Resources/Election-Polling-Resources/Margin-of-Sampling-Error-Credibility-Interval.aspx (accessed 2022).

^40^
E-mail from Tom E. Hardwicke to Jason M. Chin (Jan. 26, 2020).

^41^
See the sources at *supra* note 27.

^42^
Diamond and Mueller, *supra* note 6.

^43^
We used the 2019 list, which was the latest available when we started coding. To get a broad range of journals, we chose the top 5 on the list (Yale Law Journal, Harvard Law Review, Stanford Law Review, Columbia Law Review, and University of Pennsylvania Law Review) and the bottom 5 (Fordham Law Review, Boston College Law Review, Boston University Law Review, Cornell Law Review, and Northwestern University Law Review). We began coding in January 2021, so any issues released after that date are not included (sometimes, a year’s issue is not released until the following year); see Washington & Lee Law, W&L Journal Rankings, https://managementtools4.wlu.edu/LawJournals/ (accessed 2022).

^44^
See https://osf.io/hyk8c/ for our coded data. See https://osf.io/9q47g/ for the analytical code we used to produce the descriptive results.

^45^
Using the same method of selecting student-edited and faculty-edited journals as Chin and Zeiler, *supra* note 6.

^46^

Hardwicke *et al*., 2021, *supra* note 27.

^47^
Rose E. O’Dea *et al*., *Preferred reporting items for systematic reviews and meta-analyses in ecology and evolutionary biology: a PRISMA extension*, 96 B iol. R ev. 1695 (2021).

^48^

Hardwicke *et al*., 2021, *supra* note 27.

^49^
For an example of this approach, see Bijal Shah, *Executive (Agency) Administration*, 72 S tanford L aw R ev. 641 (2020). Although, raw data can be provided in many cases. For instance, see Oona A. Hathaway, Curtis A. Bradley and Jack L. Goldsmith, *The Failed Transparency Regime for Executive Agreements: An Empirical and Normative Analysis*, 134(2) H arv. L. R ev. 629 (2020) in which the authors digitized the data they relied on and made them available on Harvard Dataverse.

^50^
Cristina P. Sison and Joseph Glaz, *Simultaneous confidence intervals and sample size determination for multinomial proportions*, 90(429) J. A m. S tat A ssoc. 366 (1995).

^51^
Recall that some of the variables we measured are on the level of the article (i.e., article accessibility and if the article is accessible, where it is accessible; conflict of interest statement; funding statement) with all others pertaining to the first reported study within an article. For simplicity, we will refer to the units described below as “articles.” We acknowledge that there may be some bias in coding only the first reported study in that first reported studies may be different in some ways than subsequent studies in an article. However, we judged it to be unlikely that the variables we were interested in (e.g., data availability statements, preregistrations) would differ in any meaningful way across studies, and we would expect authors to adopt the same transparency approach across all studies within a single article.

^52^
We were interested in special populations because law, as an applied field, has a special interest in certain groups and stakeholders.

^53^
Matthew P. J. Ashby, *The Open-Access Availability of Criminological Research to Practitioners and Policy Makers*, 32(1) J. C rim. J us. E duc. 1 (2021); Hardwicke *et al*., 2021, *supra* note 27 at 5: “Among the 237 English-language articles, we obtained a publicly available version for 154 (65%, 95% CI = [59%, 71%]”.

^54^
Matthews and Rantanen, *supra* note 6.

^55^
See the sources at *supra* note 27.

^56^
See the sources at *supra* note 27.

^57^
Culina *et al*., *supra* note 31.

^58^
Hardwicke *et al*., *supra* note 31.

^59^
Hall and Wright, *supra* note 34 at 62. Future studies may wish to develop a way ( *a priori*) of studying the law & (economics, political science, psychology, etc.) discipline an article comes from (e.g., by reference to the journal or education background of authors) to determine if that is associated with transparency of the article’s methods.

^60^
PLOS might represent the cutting edge when it comes to disclosure of transcripts compiled in qualitative data studies (“Guidelines for qualitative data: For studies analyzing data collected as part of qualitative research, authors should make excerpts of the transcripts relevant to the study available in an appropriate data repository, within the paper, or upon request if they cannot be shared publicly. If even sharing excerpts would violate the agreement to which the participants consented, authors should explain this restriction and what data they are able to share in their Data Availability Statement. See the Qualitative Data Repository for more information about managing and depositing qualitative data.”: PLOS ONE, *Data Availability*, https://journals.plos.org/plosone/s/data-availability (accessed 2022); for best practices in data sharing, see Michelle N. Meyer, *Practical Tips for Ethical Data Sharing*, 1(1) A dv. M eth. & P ract. P sychol. S ci. 131 (2018).

^61^
Vines *et al*., *supra* note 16 (reporting that, after a request, Vines *et al*. received data for only 19% of a sample of 561 studies published between 1991 and 2011 and that the percentage received decreased over time mostly due to authors reporting that the data were lost or stored on inaccessible media); Wicherts *et al*., *supra* note 16 (reporting that, after a request, Wicherts *et al*. received data from 43% of 49 corresponding authors of papers published in 2004 by top psychology journals and that those who did not send data by six years after the initial request, which was followed by two reminders, were more likely to have reported suspect results); Tom E. Hardwicke and John P. A. Ioannidis, *Populating the Data Ark: An attempt to retrieve, preserve, and liberate data from the most highly cited psychology and psychiatry articles*, 13(8) PLOS ONE e0201856 (2018) (reporting receipt, within six months of initial request, of 32% of 111 datasets used to produce results published in highly cited psychology and psychiatry studies from 2006-2016); Wolf Vanpaemel *et al*., *Are We Wasting a Good Crisis? The Availability of Psychological Research Data after the Storm*, 1(1) C ollabra: P sychology 1 (2015) (reporting receipt, after initial request and reminders, of 38% of 394 datasets used to produce results published in four American Psychological Association journals in 2012); Michal Krawczyk and Ernesto Reuben, *(Un)Available upon Request: Field Experiment on Researchers’ Willingness to Share Supplementary Materials*, 19(3) A ccount. R es. 175 (2012) (reporting receipt of information that the authors indicated was available upon request from 44% of 200 emailed authors of studies published in 2009 by business and economics journals). In the face of such results, journals have published articles that proceed as we do, merely reporting the rate of mentions of data availability without reaching out to authors to request data. See e.g., Joshua D. Wallach, Kevin W. Boyack and John A. Ioannidis, *Reproducible research practices, transparency, and open access data in the biomedical literature, 2015-1017*, 16(11) PL oS B iol. E2006930 (2018); Hardwicke *et al*., *supra* note 27.

^62^
Mirko Gabelica, Ružica Bojčić and Livia Puljak, *Many Researchers Were Not Compliant with Their Published Data Sharing Statement: Mixed-Methods Study*, J. C linical E pidemiology (2022) https://doi.org/10.1016/j.jclinepi.2022.05.019 (reporting receipt of 7% of 1,792 datasets used to produce results published during January 2019 by BioMed Central in open access journals, in which all authors promised to provide the data upon request) *.*

^63^
Vines *et al*., *supra* note 16.

^64^
See U.S. Department of Education, Institute of Education Sciences. 2020. Policy Statement on Public Access to Data Resulting from IES Funded Grants. Available at https://sparcopen.org/wp-content/uploads/2021/01/DoEd-Policy-on-Public-Access-to-Data_IES-Funded-Grants.pdf (accessed 2022).

^65^
Meyer, *supra* note 60.

^66^
As discussed above, we saw approaches to more transparent handling of secondary data ranging from providing a detailed index of the secondary data (Shah, *supra* note 49) to digitizing the data and making it publicly available (Hathaway, Bradley and Goldsmith, *supra* note 49). Best practices documents ought to be created that explain the scenarios in which such methods are possible and desirable. See e.g., Weston, Sara J. *et al*. *Recommendations for Increasing the Transparency of Analysis of Preexisting Data Sets*, 2 A dv. M eth. & P ract. P sychol. S ci. 214 (2019). Given that users of secondary data usually modify publicly available datasets before producing results (e.g., to “clean” the data), pointing readers to the publicly available dataset is insufficient for purposes of transparency.

^67^
Tom E. Hardwicke *et al*., *Analytic reproducibility in articles receiving open data badges at the journal Psychological Science: an observational study*, 8 R. S oc. O pen sci. 201494 (2018b); Riana Minocher *et al*., *Estimating the reproducibility of social learning research published between 1955 and 2018*, 8 R. S oc. O pen sci. 210450 (2018); Hardwicke *et al*., 2018, *supra* note 27.

^68^

*Id.*

^69^
Model guidelines can be found at Center for Open Science, *The TOP Guidelines were created by journals, funders, and societies to align scientific ideals with practices*, https://www.cos.io/initiatives/top-guidelines (accessed 2022). See also PLOS ONE, *supra* note 60.

^70^
American Economic Association, Policy and Protocol on Third-Party Verifications. https://www.aeaweb.org/journals/data/policy-third-party.

^71^
Sara Schroter, *et al*., What errors do peer reviewers detect, and does training improve their ability to detect them? 101(10) J. R. S oc. M ed. 507 (2008).

^72^

*Id.*

^73^
Cortex, *Transparency and Openness Promotion (TOP) guidelines*, https://www.elsevier.com/__data/promis_misc/Cortex-TOP-author-guidelines.pdf (accessed 2022).

^74^

*Id.* PLOS, an open-access journal publishing primarily in science and medicine, will not publish studies reporting conclusions that depend solely on the analysis of proprietary data (“If proprietary data are used and cannot be accessed by others in the same manner by which the authors obtained them, the manuscript must include an analysis of publicly available data that validates the study’s conclusions so that others can reproduce the analysis and build on the study’s findings.”) See PLOS ONE, *supra* note 60. The American Economic Review requires authors to provide non-disclosable data to its data editor and/or a third-party replicator. Available at https://www.aeaweb.org/journals/data/data-code-policy (accessed 2022). On the methods front, researchers have developed new methods for disclosing data in ways that do not violate non-disclosure agreements. See Trivellore E. Raghunathan, *Synthetic Data*, 8 A nnu. R ev. S tat. A ppl. 129 (2021) (reviewing various approaches for generating and analyzing synthetic data sets that are generated to protect confidentiality).

^75^
Center for Open Science, *supra* note 69.

^76^
Moher D, et al.: The Hong Kong Principles for assessing researchers: Fostering research integrity. PLOS Biol. 2020; 18(7): e3000737. https://doi.org/10.1371/journal.pbio.3000737.

^77^

https://www.whitehouse.gov/ostp/news-updates/2023/01/11/fact-sheet-biden-harris-administration-announces-new-actions-to-advance-open-and-equitable-research/.

^78^

https://grants.nih.gov/policy/reproducibility/index.htm.

^79^
U.S. Department of Education: U.S. Department of Education Plan and Policy Development Guidance for Public Access: Improving Access to Results of Federally Funded Scientific Research. 2016. https://ies.ed.gov/funding/pdf/EDPlanPolicyDevelopmentGuidanceforPublicAccess.pdf (p. 22).

^80^
Olivier Klein *et al.*, *A Practical Guide for Transparency in Psychological Science*, 4 C ollabra: P sychology 4 (2018) 20; (2019). Sophia Crüwell *et al*., *Seven easy steps to open science: An annotated reading list*, 227 Z eitschrift für P sychologie, 237 (2019).

^81^
Jason M. Chin *et al*., *Improving the Credibility of Empirical Legal Research: Practical Suggestions for Researchers, Journals and Law Schools*, 3(1) L aw, T echnology and H umans (2021).
