# Supplementary material for: The transparency of quantitative empirical legal research published in highly ranked law journals (2018–2020): an observational study
Source: F1000Res. 2024 Mar 7;12:144. Originally published 2023 Feb 8. [Version 2] doi: 10.12688/f1000research.127563.2 (PMC10435919; doi:10.12688/f1000research.127563.2)
Supplement: Peer review history

# Reviewer report: Reviewer response for version 1

**DOI:** 10.5256/f1000research.140083.r185631

**Published:** 2023-10-12

**Authors:** Rob Heirene

**Affiliations:** 1 School of Psychology, University of Plymouth, Plymouth, UK

Many thanks for the invite to review this interesting manuscript.

 Article summary: The article reports a meta-scientific review of 300 empirical articles published in the legal field in 2019-2020). The authors aimed to determine the level of engagement with transparent/open research practices in the field and found low levels, save the accessibility of publications. The methods used are consistent with that of a systematic review; the review was preregistered; and all data, code, and materials are shared online.

 Please see my comments on each of the article sections below.

**Abstract:**

 I’m not sure whether there are any restrictions on the word count for the abstract that would preclude this, but it would be helpful to include some numerical figures in the results section (e.g., the percentage of preregistered articles) so that the readers can get a better understanding of what the authors mean by “very uncommon” etc.

**Introduction:**

 This section is well written, provides good coverage of literature related to the credibility revolution and its importance, and clearly outlines the need for the present review of empirical legal studies

 The practical benefits of engaging with transparent research practices outlined on page 4 are excellent and provide a cogent argument for data sharing etc. for researchers who may be unsure of the benefits.

 I only have one concern. On page 3, the authors state: “ *Preregistration is designed to address publication bias and questionable research practices (known in some fields as researcher degrees of freedom, p-hacking, and specification searching).*”

- I’m not familiar with the term “specification searching”, but I don’t believe researcher degrees of freedom, p-hacking, and questionable research practices are all equivalent in meaning. Researcher degrees of freedom is not inherently “bad” or indicative of QRPs, it’s just something that exists and can be exploited (the exploitation being the QRP). Similarly, while p-hacking is a type of QRP, there may be QRPs which are not a form of p-hacking. For example, a recent survey of QRPs among researchers in the Netherlands includes the following as QRPs: Making conclusions that are not sufficiently substantiated by the data, improper referencing of sources, inadequate notetaking of the research process, not submitting negative studies for publication (i.e. publication bias). While some of these may be classified as research misconduct, it is clear that many others view QRPs as more than just p-hacking.
  - Gopalakrishna *et al.* (2022 ^1^).
- The researchers could rectify this issue by providing more specific definition of publication bias and questionable research practices and/or further elaborating on each of the terms included in the parentheses. I think it’s important to provide clarity here as the authors suggest that the field of interest has not engaged well with the credibility reform and may therefore be less familiar with the terms than researchers in other fields (e.g., psychology).

**Methods:**

 On page 6 the authors link directly to their preregistration, but don’t link to their openly available data, code, and materials described in the same sentence. For consistency and the benefit of readers, I would advise directly linking to all of these resources here.

 The article search process is clearly described and its good to see the authors acknowledge the (entirely reasonable) limitations of their approach.

 It’s not entirely clear to me why the authors selected the two different samples to look at and why looking at the high-impact journals was important? Please can the authors clarify.

**Results:**

 This section is clearly presented and I have no concerns other than the minor issues below.

 How many of the reviewed studies were published in each year? Apologies if this is included somewhere and I’ve missed it.

 Figure 2: It’s very difficult to see the numbers at the far right-hand side of the C1 and C2 panels as the boxes are so small. I actually couldn’t make out what the numbers were and was unsure whether they were obscured by the border of the boxes. I know the figures are also in the table above, but I’d recommend the authors adjust the plot to put these numbers outside of the boxes and then include an arrow to point to the relevant box. When I was reproducing the authors’ analyses, I edited their script to achieve this. I have included all of the edited code relating to this figure at the end of this peer-reviewed report that the authors can use, if they wish, to achieve this. Note that I increased the size of the text labels in my edited code, since making them fit inside of the smaller boxes was no longer a concern.

**Discussion:**

 The authors provide a clear summary of their findings and how they relate to previous research in the legal field and other disciplines. A number of useful suggestions/ recommendations are provided for improving transparency and reporting practices within the field, including ways to overcome common barriers (e.g., the data access statement when privacy concerns preclude open sharing). I have no concerns or recommendations regarding this section.

**Transparency & Reproducibility:**

 I have extensively reviewed the study preregistration and the prespecified methods all appear consistent with those presented in the article, other than deviations explicitly acknowledged by the authors.

 I have also downloaded the analysis scripts and datasets from OSF and reproduced both the coding results/checks and formal analysis, including all figures and tables in the manuscript. I don’t have any concerns about the authors analysis process, but I do have a couple of suggestions for the authors to improve the reproducibility and presentation of the analyses (apologies if any of my suggestions are already well-known to the authors):

1. First of all, I commend the authors for providing their analysis scripts in a clear and intelligible format. Including a rendered R markdown document with the ability to download the underlying source code is an excellent way of sharing analysis code.
2. I ran into quite a few issues with deprecated functions in the coding checks script. I would recommend the use of a package like {groundhog} ( https://groundhogr.com/) so that anyone who tries to reproduce your code will be using the same package versions as you were when you first produced the script.
3. Relatedly, I would set the date at the top of the page to automatically be set as the date it was last rendered for full transparency (you can use this code in the YAML header of markdown and Quarto docs: date: "r format(Sys.time(), '%d %B, %Y')")
4. I would also suggest the authors include their session info at the end of the analysis document using the sessionInfo() function from the {utils} package. This will further assist with ensuring reproducibility of the script years down the line.
5. Given the benefits of using R markdown over simply sharing and R script, I would have liked to have seen the authors provide more of text description around code chunks, and break the code chunks up more so that each printed output comes from one code chunk. This would make it a lot easier to read and follow for coders and non-coders alike. The authors have done a great job of commenting the code to explain what they did, but the code is hidden and only outputs are shown – I think this is great but further necessitates the use of explanatory text before and after code chunks.
6. This may not have been possible at the time of analysis, but I would recommend using Quarto to present analysis documents in the future over RMarkdown. The syntax is practically identical and it is very similar to working with markdown, but I find the published HTML documents tend to appear neater and easier to follow, including tidier code folding options. It’s also very easy to publish the rendered document to QuartoPub ( https://quartopub.com/) so you don’t have to include a html file that has to be downloaded to be viewed on OSF (you can just include a link to a published page which is likely to be more accessible for those less familiar with coding).

To reiterate, the above are just suggestions that the authors can choose to adopt or ignore. I highly commend the authors for sharing their data files and analysis scripts in a way that allows their findings to be independently reproduced, as I have done here. This is how research should be done, but it takes time and effort and this should be recognised.

**Overall comments:**

 I hope the authors find my comments useful in revising their manuscript.

 Kind regards,

 Dr Rob Heirene ( *I sign my reviews*)

 Code (please let me know if this hasn't rendered well in the review report and I will be happy to share this via another method):

 # set up some colour palettes blue_palette <- brewer.pal(9, "Blues")[1:10] div_palette <- brewer.pal(6, "Set3")[c(1,3,4,6)] barPlot <- function(data, palette, title, legendTitle){ d <- data plt <- ggplot(data=d, aes(x=NA, y=prop, fill = var, label = label)) + geom_bar(colour = 'black', stat="identity", width = 0.25) + scale_x_discrete(breaks = NULL, expand = c(0,0)) + scale_fill_manual(values = palette, guide = guide_legend(reverse=T)) + scale_y_continuous(labels = scales::percent) + theme_minimal(base_size = 12) + theme(legend.position = "top", legend.justification='left', legend.direction='horizontal', legend.key.size = unit(1,"line"), axis.title.y=element_blank(), axis.text.y=element_blank(), axis.ticks.y=element_blank(), axis.title.x=element_blank()) + guides(fill = guide_legend(title.position = "top", nrow = 1, reverse = T)) + geom_text(data=subset(d,label != 0), size = 4, position = position_stack(vjust = 0.5)) + labs(fill=legendTitle) + ggtitle(sprintf(" %s", title)) + coord_flip(clip = 'off') + geom_text( x = 1.2, y = 1, inherit.aes = FALSE, label = sprintf("N = %i", sum(d$n)), check_overlap = TRUE, hjust = 1, size = 3 ) return(plt) }

 # build all the individual plots plt_articleAccess_student <- barPlot(d_openAccess_student, blue_palette[c(2,6)], "Article availability - Student-edited", "Status:") plt_articleAccess_peer <- barPlot(d_openAccess_peer, blue_palette[c(2,6)], "Article availability - Peer-reviewed", "Status:") plt_funding_student <- barPlot(d_funding_student, c(div_palette[c(1,4,3)], blue_palette[c(10,6)]), "Funding - Student-edited", "Statement reports:") plt_funding_peer <- barPlot(d_funding_peer, c(div_palette[c(1,4,3)], blue_palette[c(10,6)]), "Funding - Peer-reviewed", "Statement reports:") plt_dataOpen_student <- barPlot(d_dataOpen_student, blue_palette[c(2,4,6)], "Data availability - Student-edited", "Statement reports:") plt_dataOpen_peer <- barPlot(d_dataOpen_peer, blue_palette[c(2,4,6)], "Data availability - Peer-reviewed", "Statement reports:") plt_analysisOpen_student <- barPlot(d_analysisOpen_student, blue_palette[c(2,6)], "Analysis script availability - Student-edited", "Statement reports:") plt_analysisOpen_peer <- barPlot(d_analysisOpen_peer, blue_palette[c(2,6)], "Analysis script availability - Peer-reviewed", "Statement reports:") plt_materialsOpen_student <- barPlot(d_materialsOpen_student, blue_palette[c(2,6)], "Materials availability - Student-edited", "Statement reports:") plt_materialsOpen_peer <- barPlot(d_materialsOpen_peer, blue_palette[c(2,6)], "Materials availability - Peer-reviewed", "Statement reports:") plt_preregOpen_student <- barPlot(d_preregOpen_student, blue_palette[c(2,6)], "Preregistration - Student-edited", "Statement reports:") plt_preregOpen_peer <- barPlot(d_preregOpen_peer, blue_palette[c(2,6)], "Preregistration - Peer-reviewed", "Statement reports:") selected_palette <- blue_palette[c(2,4,6)] # Updated conflicts of interest plot for student edited journals: plt_conflict_student <- ggplot(data=d_conflict_student, aes(x=NA, y=prop, fill = var, label = label)) + geom_bar(colour = 'black', stat="identity", width = 0.25) + scale_x_discrete(breaks = NULL, expand = c(0,0)) + scale_fill_manual(values = selected_palette, guide = guide_legend(reverse=T)) + scale_y_continuous(labels = scales::percent) + theme_minimal(base_size = 12) + theme(legend.position = "top", legend.justification='left', legend.direction='horizontal', legend.key.size = unit(1,"line"), axis.title.y=element_blank(), axis.text.y=element_blank(), axis.ticks.y=element_blank(), axis.title.x=element_blank()) + guides(fill = guide_legend(title.position = "top", nrow = 1, reverse = T)) + geom_text(data=subset(d_conflict_student,label != 0 & prop > 0.05), size = 4, position = position_stack(vjust = 0.5)) + geom_text(data=subset(d_conflict_student,label != 0 & prop < 0.1), size = 4, position = position_stack(vjust = 155)) + # add label outside of graph labs(fill="Statement reports:") + ggtitle(sprintf(" %s", "Conflicts of interest - Student-edited")) + coord_flip(clip = 'off') + geom_text( x = 1.2, y = 1, inherit.aes = FALSE, label = sprintf("N = %i", sum(d$n)), check_overlap = TRUE, hjust = 1, size = 3 ) + # Add arrow geom_segment(aes(x = 1, y = 1.03, xend = 1, yend = 1.001), arrow = arrow(length = unit(0.03, "npc")), size = 1.1) # Updated conflicts of interest plot for peer reviewed journals: plt_conflict_peer <- ggplot(data=d_conflict_peer, aes(x=NA, y=prop, fill = var, label = label)) + geom_bar(colour = 'black', stat="identity", width = 0.25) + scale_x_discrete(breaks = NULL, expand = c(0,0)) + scale_fill_manual(values = selected_palette, guide = guide_legend(reverse=T)) + scale_y_continuous(labels = scales::percent) + theme_minimal(base_size = 12) + theme(legend.position = "top", legend.justification='left', legend.direction='horizontal', legend.key.size = unit(1,"line"), axis.title.y=element_blank(), axis.text.y=element_blank(), axis.ticks.y=element_blank(), axis.title.x=element_blank()) + guides(fill = guide_legend(title.position = "top", nrow = 1, reverse = T)) + geom_text(data=subset(d_conflict_peer,label != 0 & prop > 0.05), size = 4, position = position_stack(vjust = 0.5)) + geom_text(data=subset(d_conflict_peer,label != 0 & prop < 0.1), size = 4, position = position_stack(vjust = 155)) + # add label outside of graph labs(fill="Statement reports:") + ggtitle(sprintf(" %s", "Conflicts of interest - Student-edited")) + coord_flip(clip = 'off') + geom_text( x = 1.2, y = 1, inherit.aes = FALSE, label = sprintf("N = %i", sum(d$n)), check_overlap = TRUE, hjust = 1, size = 3 ) + # Add arrow geom_segment(aes(x = 1, y = 1.03, xend = 1, yend = 1.002), arrow = arrow(length = unit(0.03, "npc")), size = 1.1)

 Figure 2

Is the work clearly and accurately presented and does it cite the current literature?

Yes

If applicable, is the statistical analysis and its interpretation appropriate?

Yes

Are all the source data underlying the results available to ensure full reproducibility?

Yes

Is the study design appropriate and is the work technically sound?

Yes

Are the conclusions drawn adequately supported by the results?

Yes

Are sufficient details of methods and analysis provided to allow replication by others?

Yes

Reviewer Expertise:

Psychology; Addiction; Scientific reform

I confirm that I have read this submission and believe that I have an appropriate level of expertise to confirm that it is of an acceptable scientific standard.

## References

[1] : Prevalence of questionable research practices, research misconduct and their potential explanatory factors: A survey among academic researchers in The Netherlands. *PLoS One* .2022;17(2) : 10.1371/journal.pone.0263023 e0263023 10.1371/journal.pone.0263023 35171921 PMC8849616

# Response

**Published:** 2023-12-05

**Authors:** Jason Chin

**Affiliations:** Australian National University, Australia

Thank you Rob for the feedback and helpful code!

 We were waiting for the final review to begin revisions. We expect to post them in mid-January 2024. - Jason et al

# Reviewer report: Reviewer response for version 1

**DOI:** 10.5256/f1000research.140083.r185632

**Published:** 2023-08-17

**Authors:** Stefanie Mueller

**Affiliations:** 1 Leibniz Institute for Psychology (ZPID), Trier, Germany

The manuscript describes the adoption of transparency practices for studies in the field of legal research. The studies were published in either faculty-edited journals or in student-edited journals. Student-edited journals were selected based on their Washington & Lee ranking (which I assume is a well-known source in the legal community); faculty-edited journals were selected based on their impact factor as reported in the Web of Science”law” database.

 Within these journals, the authors’ goal was to select studies that analyzed quantitative data. To this end, articles were pre-selected using a pilot-tested search string, then the resulting lists of articles were randomized and manually screened by one of the authors until the pre-specified sample size of 150 for each list was reached. A pre-existing coding scheme was used to assess the study characteristics, with a focus on features of transparency (e.g. data availability). The authors report the results descriptively using absolute numbers and percentages. They also report confidence intervals calculated using a method appropriate for their data. They find generally low transparency for most features except for article accessibility. 

 The manuscript is very well written and models the standards of transparency that the authors (and I) would like to see in (quantitative) research. The background, procedure, and results were easy to follow while explained in detail. The methods seem appropriate to me. All in all, I have only a few and very minor suggestions for improvement. 

- Typo on page 7, under “sample”: “150 faculty-edited journals from the 25 journals” → “150 faculty-edited articles from the 25 journals”.
- Fig 1: I suggest using a sans serif font for better readability.
- Table 2: I suggest using the same darker background color for the row “Studies in student-edited journals” and “Studies in faculty-edited journals” as for all as it helps the reader to classify the main categories
- Fig 2 and 3: The figures are redundant to the data presented in the table and currently do not add value (in my honest opinion). I’d suggest either dropping them completely or re-organizing them a bit so that they become clearer. (e.g. increase distance between the bar graph and the heading of the next panel below, use the same bar width for Fig 2 as for Fig 3; alternatively: re-organize the plot by placing all the bar graphs in the right column and the label student-/faculty-edited in the rows, that would aid the visual comparison between student-/faculty-edited and allows you to reduce the legends; then, arrange the bar graphs in pairs according to the dimensions displayed, i.e., smaller distance between members of one pair, larger distance to the next pair).

Is the work clearly and accurately presented and does it cite the current literature?

Yes

If applicable, is the statistical analysis and its interpretation appropriate?

Not applicable

Are all the source data underlying the results available to ensure full reproducibility?

Yes

Is the study design appropriate and is the work technically sound?

Yes

Are the conclusions drawn adequately supported by the results?

Yes

Are sufficient details of methods and analysis provided to allow replication by others?

Yes

Reviewer Expertise:

Experimental Psychology, Meta-Science

I confirm that I have read this submission and believe that I have an appropriate level of expertise to confirm that it is of an acceptable scientific standard.

# Response

**Published:** 2023-12-05

**Authors:** Jason Chin

**Affiliations:** Australian National University, Australia

Thank you Stefanie for these helpful comments. 

 We were waiting for the final review to begin revisions. We expect to post them in mid-January 2024. - Jason et al

# Reviewer report: Reviewer response for version 1

**DOI:** 10.5256/f1000research.140083.r162735

**Published:** 2023-02-27

**Authors:** Antica Culina

**Affiliations:** 1 Netherlands Institute of Ecology, NIOO-KNAW, Wageningen, The Netherlands

The MS uses articles published in the field of empirical legal research to study the occurrence of several practices related to transparent research and credibility revolution – such as preregistration, open data and protocols, or open access to publication. These appear to be very low in empirical legal research. As the first that looked into how common these practices are in the field, this paper can serve as a benchmark for any further improvements in the field. Further, it can serve as a encouragement to improve upon the situation.

 The study is well presented, easy to follow, and applies the transparent practices in its design, reporting and conduct. With this it can also be a great example of how studies in the field of empirical legal research can be done. I really enjoyed reading this work.

 My suggestion involve:

1. In the intro, I suggest to first present the benefits of open practices that are not connected with e.g. spotting errors and similar (these usually go less well with researchers). E.g. first emphasize the benefits to research/public as data and codes or methods are available so other can use them for different type of research, evidence synthesis etc, which all bring much higher gain from conducted research. And then I would always mention error checking and similar after these more `positive` outcomes.
2. I am unclear as of how well the general readership of the journal will be familiar with some of the terms, e.g. replication failure. If readership is unlikely to know about these, maybe provide some more background on the transparent practices in the intro, or provide a table with the definitions (e.g. open data, open materials, open code, preregistration…)
3. Have high impact journals been chosen so there is a higher likelihood of detecting existence of some of the transparent practices? This is not clear from what is currently represented in the MS.
4. Before the method section, or at its beginning, the reader needs to know what exactly is this MS after? What exact practices are examined. Up to that point this is vague – rather, only some parts of what will be considered are mentioned (e.g. transparent practices) but it is not clear what this exactly entails.
5. I remain unclear on how the search string was derived to. Some of the terms seem quite random. E.g. content analysis? Is the sting truly providing an unbiased set of studies in the field? (apart from not finding studies without an abstract).
6. Introduction does not say much about general state of journal and funders policies on transparency in the field. Is it something that is becoming required? Do they follow some other, maybe more progressive fields?
7. Table 4 – why were these examples selected?
8. Table 4 – for Culina et al, 79% articles had available data, so 21% did not have data available. Also, note that Culina et al did not look into Data or Analysis Codes Availability statements, but rather whether data/analysis were available somewhere, regardless of whether they were mentioned as such.
9. Can ` Where do we go from here? ` paragraph be separated as a subsection if journal allows? I think this section is vary important, as the current MS sets the stage by providing the evidence that the field is not transparent.
10. In the call for change in practices, I would add a few points

1. Include funders: they also set standards for the work they fund, e.g. by setting data sharing policy for funded research. Funders (and institutions) should also help researchers when researchers want to apply transparent practices (e.g. by providing data stewards).
2. Rewards and assessment system must also change. E.g. DORA declaration is a great example of rewarding practices other than publishing in high impact journals. If more research / academic institutions and funders would apply an altered assessment system, open practices would likely become more common
3. A step further for journals is once they have a policy, that they engage data editors, who check if the material submitted are indeed contain the information they state they contain.

Is the work clearly and accurately presented and does it cite the current literature?

Yes

If applicable, is the statistical analysis and its interpretation appropriate?

Not applicable

Are all the source data underlying the results available to ensure full reproducibility?

Yes

Is the study design appropriate and is the work technically sound?

Yes

Are the conclusions drawn adequately supported by the results?

Yes

Are sufficient details of methods and analysis provided to allow replication by others?

Yes

Reviewer Expertise:

meta research , evolutionary ecology

I confirm that I have read this submission and believe that I have an appropriate level of expertise to confirm that it is of an acceptable scientific standard.

# Response

**Published:** 2023-12-05

**Authors:** Jason Chin

**Affiliations:** Australian National University, Australia

Thanks so much Antica - we were waiting for the final review to begin revisions. We expect to post them in mid-January 2024. - Jason et al
